# Bioinformatics analysis of differentially expressed genes and identification of an miRNA–mRNA network associated with entorhinal cortex and hippocampus in Alzheimer’s disease

**DOI:** 10.1186/s41065-021-00190-0

**Published:** 2021-07-09

**Authors:** Haoming Li, Linqing Zou, Jinhong Shi, Xiao Han

**Affiliations:** 1 Department of Human Anatomy, Institute of Neurobiology, Medical School of Nantong University, 19 Qixiu Road, Nantong, 226001 Jiangsu China; 2 Key Laboratory of Neuroregeneration of Jiangsu and Ministry of Education, Co-Innovation Center, Neuroregeneration of Nantong University, Nantong, 226001 Jiangsu China

**Keywords:** Alzheimer’s disease, Entorhinal cortex, Hippocampus, Differentially expressed genes, microRNAs

## Abstract

**Background:**

Alzheimer’s disease (AD) is a fatal neurodegenerative disorder, and the lesions originate in the entorhinal cortex (EC) and hippocampus (HIP) at the early stage of AD progression. Gaining insight into the molecular mechanisms underlying AD is critical for the diagnosis and treatment of this disorder. Recent discoveries have uncovered the essential roles of microRNAs (miRNAs) in aging and have identified the potential of miRNAs serving as biomarkers in AD diagnosis.

**Methods:**

We sought to apply bioinformatics tools to investigate microarray profiles and characterize differentially expressed genes (DEGs) in both EC and HIP and identify specific candidate genes and pathways that might be implicated in AD for further analysis. Furthermore, we considered that DEGs might be dysregulated by miRNAs. Therefore, we investigated patients with AD and healthy controls by studying the gene profiling of their brain and blood samples to identify AD-related DEGs, differentially expressed miRNAs (DEmiRNAs), along with gene ontology (GO) analysis, KEGG pathway analysis, and construction of an AD-specific miRNA–mRNA interaction network.

**Results:**

Our analysis identified 10 key hub genes in the EC and HIP of patients with AD, and these hub genes were focused on energy metabolism, suggesting that metabolic dyshomeostasis contributed to the progression of the early AD pathology. Moreover, after the construction of an miRNA–mRNA network, we identified 9 blood-related DEmiRNAs, which regulated 10 target genes in the KEGG pathway.

**Conclusions:**

Our findings indicated these DEmiRNAs having the potential to act as diagnostic biomarkers at an early stage of AD.

**Supplementary Information:**

The online version contains supplementary material available at 10.1186/s41065-021-00190-0.

## Introduction

Alzheimer’s disease (AD) is the most frequent cause of dementia, accounting for 60–80% of all such cases [1]. An estimated 47 million people were affected by dementia in 2015, but this number is projected to triple by 2050 [2]. AD is an age-related progressive neurodegenerative disorder, and the most common type is the late-onset, also referred to as sporadic AD, which is defined as AD with an age-onset > 65 years old, and is ascribed to a complex combination of an individual's genes, environment, and lifestyle habits. Whereas, the early-onset AD, also called familial AD (FAD), occurs at onset ages ranging from 30–65 years old, and its rarely hereditary involving the amyloid precursor protein, presenilin-1 (PS1), presenilin-2 (PS2) and apolipoprotein E (APOE) ɛ4 allele genes [3, 4]. More than 90% of AD cases are sporadic, characterized by late-onset and it is driven by a complex interplay between genetic and environmental factors, approximately 70% of sporadic cases are risk associated with genetic factors involvement [5]. For example, the APOE gene, one of the famous AD-related genes, has three variants (ε2, ε3, ε4), and APOE ε4 is considered as the single highest risk for sporadic AD, whereas APOE ε2 is associated with decreased risk of AD [6].

The development of intraneuronal lesions at vulnerable brain sites is central to AD. The main lesions include hyperphosphorylated tau protein, neurofibrillary tangles (NFTs) in cell bodies, and neuropil threads in neuronal processes [7–9]. Braak staging is extensively used to classify the degree of semiquantitative measure of NFTs pathology in the brain autopsy of AD. The pathology is performed using a modern silver technique and evaluated the development and the topographic expansion of the AD lesions [10]. In recent years, a revised procedure is used to facilitate the uniform application of the staging procedure, which is processed by immunostaining for hyperphosphorylated tau protein AT8 [11]. The pathology of the progression of AD begins in structures of the entorhinal cortex (EC) and hippocampus (HIP) in the prodromal stage (Braak staging I-II). In this stage, the lesions mainly intrude into transentorhinal, entorhinal region, and hippocampal Ammon's horn (CA1/CA2). After that, in the early-moderate stage (Braak staging III-IV), the lesions invade into the limbic area and the mature neocortex. In the moderate-late stage (Braak staging V-VI), the neocortical pathology fully extends into the motor and sensory regions of neocortical regions [10, 11].

Recent discoveries indicate that comprehensive bioinformatical analyses could provide novel therapeutic targets participating in the pathology of AD [12–15]. However, the diagnosis using AD biomarkers are impossible to detect at early stages of AD, making the identification of early and noninvasive biomarkers for AD still very challenging. MicroRNAs (miRNAs) are a class of small non-coding RNAs (ncRNAs) that acts as important post-transcriptional regulators of gene expression by targeting mRNAs. In the last decade, miRNAs-mediated regulation has signified a new target of therapeutic prospects [16], with numerous pieces of evidence undoubtedly showing the involvement of miRNAs in both the pathophysiology and pharmacotherapy of neurodegenerative disorders [17], and also uncover the crucial roles of miRNAs during aging, with the identification of more miRNAs as biomarkers in the diagnosis of AD [12, 18].

Considering the lesions begins in EC and HIP at the early stage of AD, and circulating miRNAs in biofluids could be detected readily and acted as biomarkers in AD diagnosis [19, 20]. In this study, we applied bioinformatics tools to investigate microarray profiles and identified differentially expressed genes (DEGs) in both EC and HIP, and crucial genes and pathways were analyzed further. In addition, we presumed that these DEGs could be dysregulated by miRNAs in blood of patients with AD; thus, an AD-specific miRNA–mRNA network was constructed to identify differentially expressed miRNAs (DEmiRNAs) and to explore their involvement in AD.

## Materials and methods

### Microarray expression profiling and differentially expressed genes screening

**Fig. 1:**
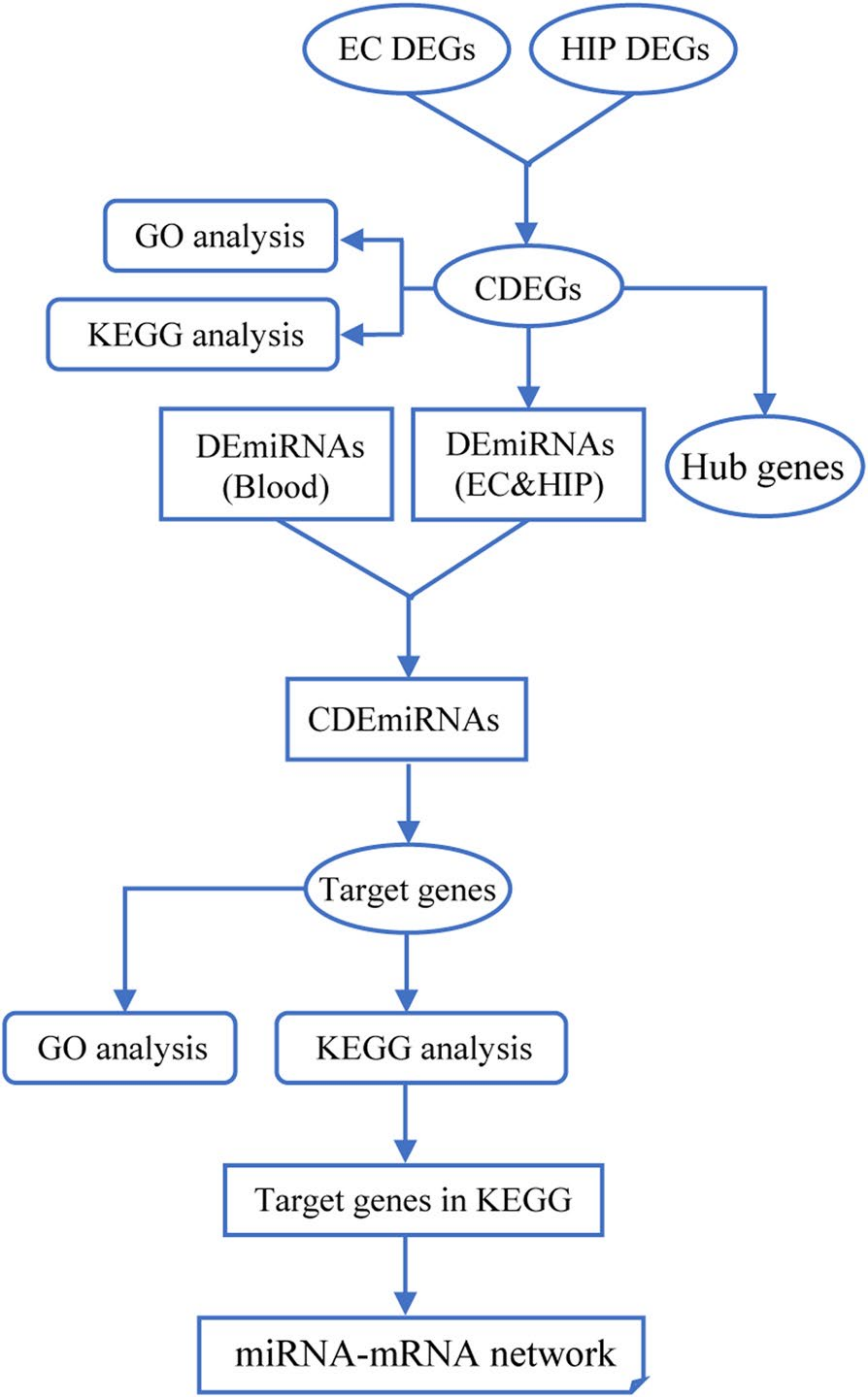
The schematic diagram of this study

The workflow of this study was described as the schematic diagram in Fig. 1. Briefly, a dataset GSE5281 containing mRNA expression profiles of EC and HIP samples was downloaded from the Gene Expression Omnibus (GEO, https://www.ncbi.nlm.nih.gov/geo) [21, 22]. AD brain samples were obtained from patients with clinically and neuropathologically diagnosed late-onset AD (15 males and 18 females) with a mean age of 79.9 ± 6.9 years [22]. Normal brain samples were from individuals classified as neurologically normal (10 males and 4 females) with a mean age of 79.8 ± 9.1 years [21]. In total, 10 EC and 10 HIP AD samples along with 13 EC and 13 HIP normal brain samples were used in this study. The raw data was normalized and utilized to screen DEGs by using the Limma package [23] in R software (https://cran.r-project.org/). A |Log_2_FC(Fold Change)|≥ 1 and adjusted to a *p*-value < 0.05 were considered as statistically significant. The miRNAs dataset was derived from the microarray experiment by Petra Ledinger et al. [24]. In this dataset, the expression of miRNAs in peripheral blood of 48 patients with AD and 22 unaffected controls was analyzed, and 140 significantly dysregulated mature miRNAs were displayed.

### Heatmaps and Venn diagram drawing

The pheatmap package (https://cran.r-project.org/web/packages/pheatmap/) in R software was used to plot the heatmap of DEGs**.** The intersection part of DEGs or DEmiRNAs was plotted as a Venn diagram by using the VennDiagram package in R software (https://cran.r-project.org/web/packages/VennDiagram) or TBtools software [25].

### Gene ontology and pathway enrichment analysis

Gene ontology (GO) enrichment analysis and KEGG pathway analysis were used to evaluate the function and biological processes of DEGs. GO and KEGG analyses were performed by the online website Database for Annotation, Visualization, and Integrated Discovery (DAVID, https://david.ncifcrf.gov/) [26, 27]. Gene counts ≥ 3 and *p*-value < 0.05 were set as the screening threshold.

### Protein–protein interaction network construction and hub genes identification

We evaluated the functional associations and the interactive relationships among the DEGs or target genes of DEmiRNAs by uploading all the genes into the online STRING database (https://string-db.org/) [28] for the prediction of protein–protein interactions (PPI). Interactions with a combined score > 0.4 were considered significant. The PPI network of DEGs was visualized using Cytoscape software [29].

The hub genes were extracted by cytoHubba plugin in Cytoscape software [30]. A total of 12 topological analyses were provided by this plugin, and three most widely used analysis methods were used in our work, as previously report [31], including maximal clique centrality (MCC), the density of maximum neighborhood component (DMNC), and maximum neighborhood component (MNC). The identified top 20 hub genes were selected using each method, and the overlapping genes were determined as hub genes using the Venn diagrams by TBtools software [25].

The significant modules in the PPI network were identified by using molecular complex detection (MCODE) plugin in Cytoscape software, which could find clusters based on their topology to recognize highly interconnected regions in a network [32]. The parameters of MCODE were set as previously reported, as follows: MCODE score ≥ 4, degree cut-off = 2, node score cut-off = 0.2, max depth = 100, k-core = 2 [33].

### miRNA-target genes prediction and miRNA–mRNA network construction

The potential miRNAs for the selected hub genes were predicted using online miRNet software (http://www.mirnet.ca/). miRNet provides a comprehensive analysis of high-quality miRNA-target interaction data based on 11 different miRNA databases, including TarBase, miRTarBase, miRecords, miRanda, miR2Disease, HMDD, PhenomiR, SM2miR, PharmacomiR, EpimiR, and starBase [34]. The miRNA–mRNA interaction network was analyzed and constructed using Cytoscape software [29].

### Statistical analysis

All statistical analyses were performed in Graphpad Prism 8 software (GraphPad Software Inc., San Diego, CA, USA). Comparison of target genes was performed using one-sample Student's *t*-tests for parametric data, whereas one-sample Wilcoxon tests were used to measure nonparametric data. The results were considered significant when *p* < 0.05.

## Results

### Identification of common DEGs in EC and HIP

**Fig. 2:**
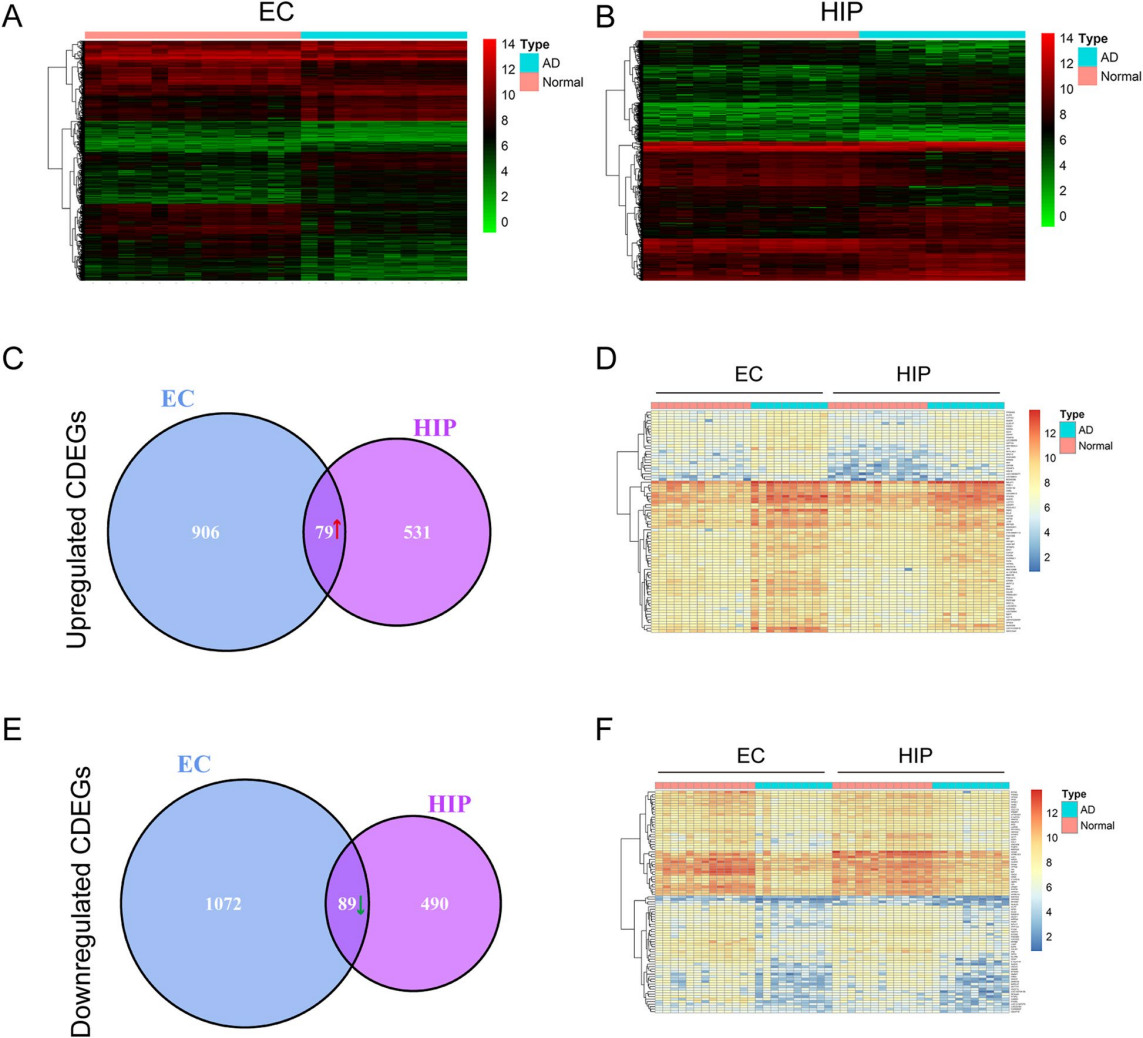
A total of 2146 differentially expressed genes (DEGs) were identified in the EC samples (**A**), and 1189 DEGs were identified in the HIP samples (**B**). Then 79 upregulated common DEGs (CDEGs) were found in EC and HIP (**C**), the heatmap (**D**) showed the expression level of these upregulated CDEGs. Also, 89 downregulated CDEGs (**E**) were found in both EC and HIP, the heatmap (**F**) revealed the expression level of these downregulated CDEGs

A total of 2146 DEGs were identified in the EC samples, including 985 upregulated DEGs and 1161 downregulated DEGs (Fig. 2A and Supplementary Data.1 ). A total of 1189 DEGs were identified in the HIP sample, including 610 upregulated DEGs and 579 downregulated DEGs (Fig. 2B and Supplementary Data.2 ). After identification of the common DEGs (CDEGs) in EC and HIP, we found 168 CDEGs in EC and HIP (Supplementary Data.3 ), including 79 upregulated CDEGs (Fig. 2 C, D) and 89 downregulated CDEGs (Fig. 2 E, F). The heatmaps of upregulated CDEGs (Fig. 2 D) and downregulated CDEGs (Fig. 2 F) further presented the expression level of these CDEGs in EC and HIP.

### Functional annotation of common DEGs

**Fig. 3:**
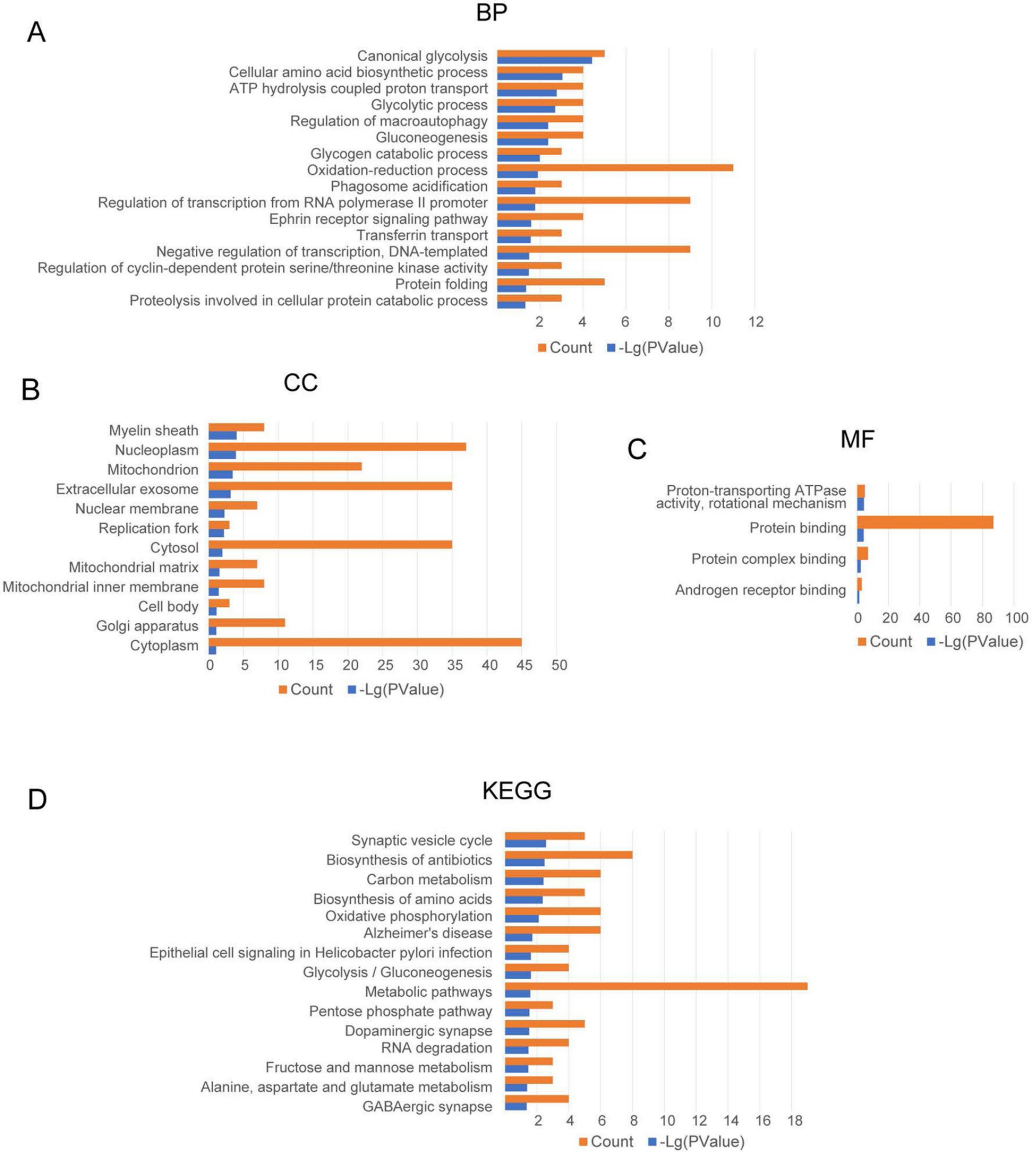
Significant common differentially expressed genes (CDEGs) were enriched in the biological process (BP) (**A**), cellular component (CC) (**B**), and molecular function (MF) (**C**) terms. KEGG pathway analysis showed that the CDEGs were mainly enriched in the synaptic vesicle cycle, biosynthesis of antibiotics, carbon metabolism, biosynthesis of amino acids, oxidative phosphorylation, and Alzheimer's disease (**D**)

The GO enrichment analysis was conducted three items. In the biological process (BP) category, the GO terms were mainly associated with canonical glycolysis, cellular amino acid biosynthetic process, ATP hydrolysis-coupled proton transport, glycolytic process, regulation of macroautophagy, and gluconeogenesis (Fig. 3A). In the cellular component (CC) group, the GO terms were mainly associated with myelin sheath, nucleoplasm, mitochondrion, extracellular exosome, nuclear membrane, and replication fork (Fig. 3B). In the molecular function (MF) group, the GO terms mainly involved in proton-transporting ATPase activity, protein binding, protein complex binding, and androgen receptor binding (Fig. 3C). KEGG pathway enrichment analysis showed the CDEGs were mainly enriched in synaptic vesicle cycle, biosynthesis of antibiotics, carbon metabolism, biosynthesis of amino acids, oxidative phosphorylation, and AD (Fig. 3D).

### Hub gene analysis and PPI network construction

To get hub genes of CDEGs, the cytoHubba plugin in Cytoscape software was used to extract hub genes. The cytoHubba plugin can score and rank nodes in a network based on different algorithms [30]. A total of 12 scoring methods were provided in the cytoHubba plugin to analyze a network, and the top-ranked nodes of a particular scoring method were identified as hub genes. In this work, we identified the 20 top-ranked nodes as hub genes using the three most widely used analysis algorithms, namely MCC, DMNC, and MNC. Then, the 15 overlapping hub genes were determined (Fig. S1A), including PYGB, GPI, PFKFB3, ATP5C1, ENO1, ATP5B, EIF3G, ATP6V1H, PMPCA, ALDOC, ME3, ATP6V0D1, ATP6V1E1, NDUFV1, and PFKM. Then, a PPI network of these hub genes were constructed using the online STRING database and visualized using Cytoscape software (Fig. S1B). EIF3G didn’t interact with other factors in STRING database, so EIF3G was excluded in this PPI network.

**Fig. 4:**
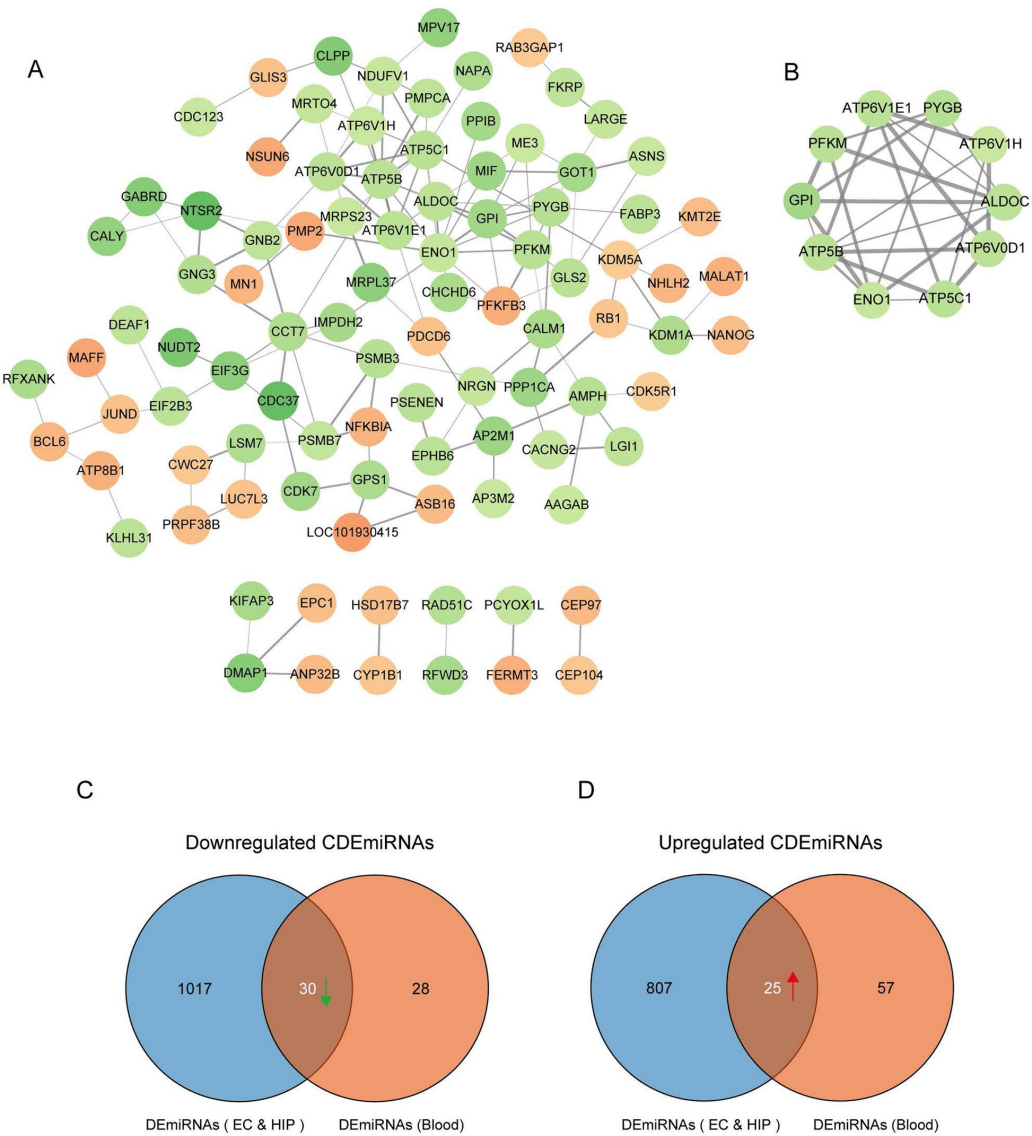
A protein–protein interaction (PPI) network of all common differentially expressed genes (CDEGs) included 96 nodes and 144 edges (**A**). One significant module was selected using the MCODE plugin, this module consisted of 10 nodes and 25 edges (**B**). Using miRNet online tools, 1047 predicted differentially expressed microRNAs (DEmiRNAs) of 79 upregulated CDEGs (**C**), and 832 predicted DEmiRNAs of 89 downregulated CDEGs (**D**) were identified in EC and HIP samples. The DEmiRNAs from EC and HIP were intersected with the DEmiRNAs from blood samples, 55 common DEmiRNAs (CDEmiRNAs) were further identified, including 30 downregulated CDEmiRNAs (**C**) and 25 upregulated CDEmiRNAs (**D**)

Next, we explored the significant modules and hub genes by using the MCODE plugin in Cytoscape. After constructing of a PPI network of all CDEGs, which includes 96 nodes and 144 edges (Fig. 4A), one significant module from the PPI network was screened (Fig. 4B). This module consisted of 10 nodes and 25 edges, and all nodes within this module also existed in the hub genes, which were identified by the cytoHubba plugin. We inferred that these 10 nodes acted as key hub genes in all of the CDEGs (Fig. 4B and Table 1).

**Table 1:** Ten key hub genes were screened by using cytoHubba and MCODE plugins in Cytoscape software. All these genes were involved in energy metabolism. GPI, PYGB, PFKM, ALDOC, and ENO1 were glycolytic metabolism-related genes. ATP5C1, ATP5B, ATP6V1E1, ATP6V0D1, and ATP6V1H were ATP metabolism-related genes

Symbols	Full Names	Gene IDs
GPI	Glucose-6-phosphate isomerase	2821
PYGB	Glycogen phosphorylase B	5834
PFKM	Phosphofructokinase, muscle	5213
ATP5C1 (ATP5F1C)	ATP synthase F1 subunit gamma	509
ATP5B (ATP5F1B)	ATP synthase F1 subunit beta	506
ATP6V1E1	ATPase H + transporting V1 subunit E1	529
ALDOC	Aldolase, fructose-bisphosphate C	230
ATP6V0D1	ATPase H + transporting V0 subunit d1	9114
ENO1	Enolase 1	2023
ATP6V1H	ATPase H + transporting V1 subunit H	51,606

### Prediction of miRNAs and identification of common DEmiRNAs

Further, we explored the predicted miRNAs of CDEGs in EC and HIP using miRNet online tools [34]. We found 1047 predicted differentially expressed microRNAs (DEmiRNAs) of the 79 upregulated CDEGs (Fig. 4C, Supplementary Data. 4 ), and 832 predicted DEmiRNAs of the 89 downregulated CDEGs (Fig. 4D, Supplementary Data. 5 ). Also, 140 specifically dysregulated DEmiRNAs (58 downregulated and 82 upregulated DEmiRNAs) in blood samples of patients with AD were identified and used in our work, according to the report by Petra Leidingger et al. (Supplementary Data. 6 ) [24]. The DEmiRNAs from EC and HIP were intersected with the DEmiRNAs from blood samples; the common DEmiRNAs (CDEmiRNAs) were identified. In total, we found 55 CDEmiRNAs (Supplementary Data. 7 ), including 30 downregulated CDEmiRNAs (Fig. 4C) and 25 upregulated CDEmiRNAs (Fig. 4D).

### Identification of 10 target genes in the KEGG pathway and construction of an miRNA–mRNA network

**Fig. 5:**
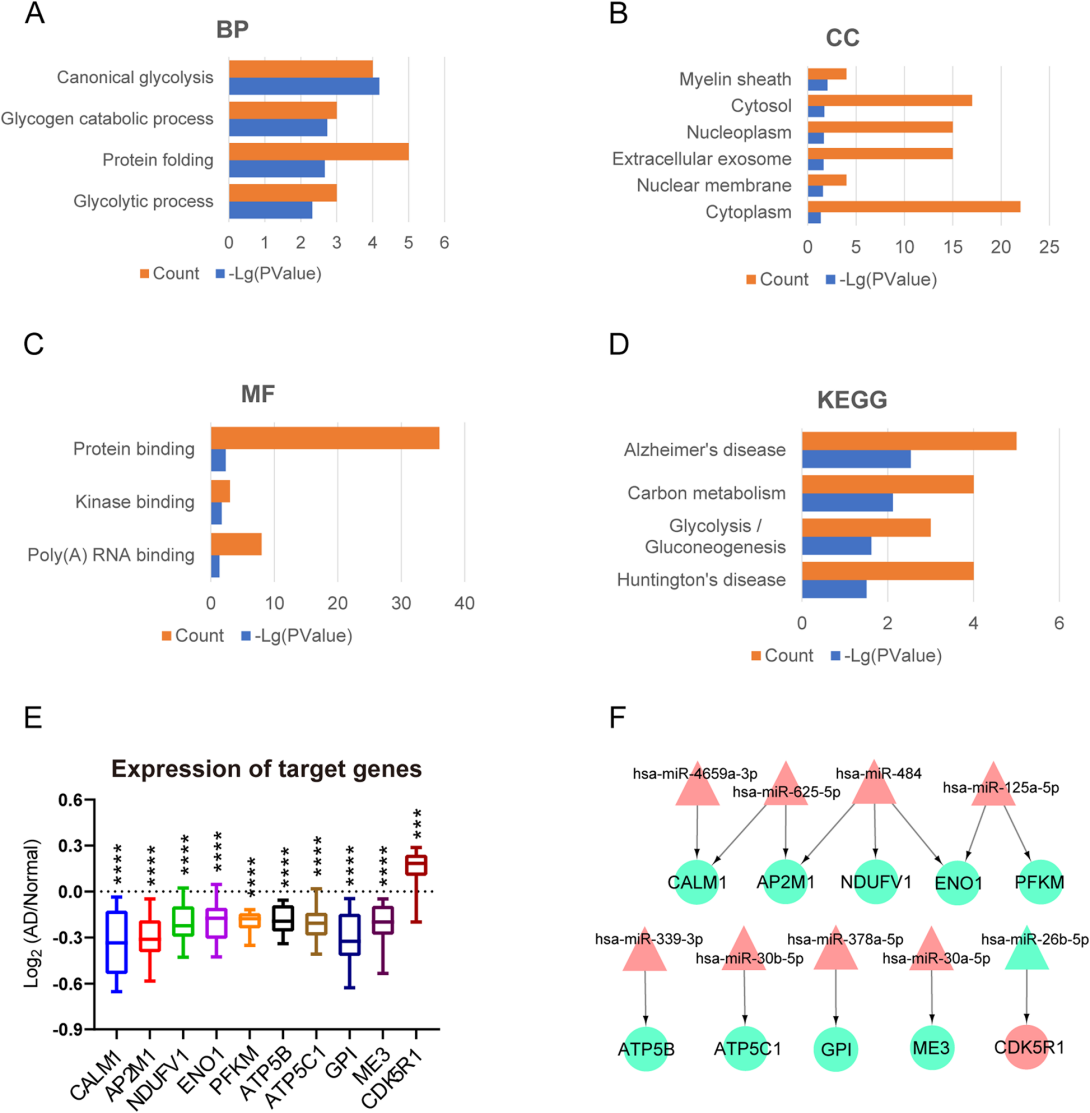
Significant biological process (BP) (**A**), cellular component (CC) (**B**), and molecular function (MF) (**C**) terms enriched in target genes of 55 common differentially expressed microRNAs (CDEmiRNAs). KEGG pathway analysis showed the target genes were mainly enriched in Alzheimer's disease, carbon metabolism, glycolysis/gluconeogenesis, and Huntington's disease (**D**). 10 target genes were found in the KEGG pathway analysis, including CALM1, AP2M1, NDUFV1, ENO1, PFKM, ATP5B, ATP5C1, GPI, ME3, and CDK5R1 (**E**). CDK5R1 was an increased gene, the other 9 target genes were all decreased its expression in AD samples, *** *p* < 0.001, **** *p* < 0.0001. An miRNA–mRNA network of target genes discovered that 9 CDEmiRNAs were involved in the regulation of these 10 target genes (**F**). The nine downregulated target genes were regulated by hsa-miR-4659a-3p, hsa-miR-625-5p, hsa-miR-484, hsa-miR-125a-5p, hsa-miR-339-3p, hsa-miR-30b-5p, hsa-miR-378a-5p, and hsa-miR-30a-5p, and the increased CDK5R1 was regulated by hsa-miR-26b-5p, red labels represented upregulated miRNAs and target genes, and green labels represented downregulated miRNAs and target genes

Finally, we identified further, the target genes of the 55 CDEmiRNAs. In total, we found 59 target genes of all CDEmiRNAs, including 30 upregulated target genes of the 30 downregulated CDEmiRNAs (Supplementary Data. 8 ), and 29 downregulated target genes of the 25 upregulated CDEmiRNAs (Supplementary Data. 9 ). GO enrichment analysis, and KEGG pathway analysis of 59 target genes was processed further. In the BP category, the GO term was mainly associated with canonical glycolysis, glycogen catabolic process, protein folding, and glycolytic process (Fig. 5A). In the CC category, the GO term was focused on the myelin sheath, cytosol, nucleoplasm, extracellular exosome, nuclear membrane, cytoplasm (Fig. 5B). In MF, the GO term was associated with protein binding, kinase binding, and poly(A) RNA binding (Fig. 5C). The KEGG pathway enrichment analysis showed that the target genes mainly enriched in AD, carbon metabolism, glycolysis/gluconeogenesis, and Huntington's disease (Fig. 5D).

We found 10 target genes involved in the KEGG pathway, including CALM1, AP2M1, NDUFV1, ENO1, PFKM, ATP5B, ATP5C1, GPI, ME3, CDK5R1 (Table 2). Among these 10 target genes, ENO1, PFKM, ATP5B, ATP5C1, and GPI also belonged to the 10 key hub genes (Fig. 4B and Table 1). Subsequently, we measured the expression level of these 10 target genes in the AD samples, and each gene was compared with the mean expression level in the control individuals. We found that, except CDK5R1, which was an increased gene, the other 9 target genes all decreased in expression in the AD samples than that in the control samples (Fig. 5E). Moreover, we found that 9 CDEmiRNAs were involved in the regulation of these 10 target genes (Table 3). For better visualization, we constructed the miRNA–mRNA network of target genes and CDEmiRNAs using Cytoscape software (Fig. 5F). The 9 downregulated target genes were regulated by hsa-miR-4659a-3p, hsa-miR-625-5p, hsa-miR-484, hsa-miR-125a-5p, hsa-miR-339-3p, hsa-miR-30b-5p, hsa-miR-378a-5p, and hsa-miR-30a-5p, while the increased gene, CDK5R1, was regulated by hsa-miR-26b-5p.

**Table 2:** Ten target genes were involved in the KEGG pathway, including CALM1, AP2M1, NDUFV1, ENO1, PFKM, ATP5B, ATP5C1, GPI, ME3, CDK5R1, which were mainly enriched in Alzheimer's disease, carbon metabolism, glycolysis/gluconeogenesis, and Huntington's disease. The bold label (ENO1, PFKM, ATP5B, ATP5C1, and GPI) meant these genes also belonged to the key hub genes

Symbols	Full Names	Gene IDs
CALM1	Calmodulin 1	801
AP2M1	Adaptor related protein complex 2 subunit mu 1	1173
NDUFV1	NADH:ubiquinone oxidoreductase core subunit V1	4723
**ENO1**	Enolase 1	2023
**PFKM**	Phosphofructokinase, muscle	5213
**ATP5B** (ATP5F1B)	ATP synthase F1 subunit beta	506
**ATP5C1** (ATP5F1C)	ATP synthase F1 subunit gamma	509
**GPI**	Glucose-6-phosphate isomerase	2821
ME3	Malic enzyme 3	10,873
CDK5R1	Cyclin dependent kinase 5 regulatory subunit 1	8851

**Table 3:** Nine common differentially expressed miRNAs (CDEmiRNAs), which were predicted to regulate 10 target genes in the KEGG pathway

Accession	miRNAs IDs	Target genes symbols
MIMAT0004702	hsa-miR-339-3p	ATP5B
MIMAT0002174	hsa-miR-484	NDUFV1
MIMAT0000420	hsa-miR-30b-5p	ATP5C1
MIMAT0019727	hsa-miR-4659a-3p	CALM1
MIMAT0003294	hsa-miR-625-5p	CALM1
MIMAT0000731	hsa-miR-378a-5p	GPI
MIMAT0000087	hsa-miR-30a-5p	ME3
MIMAT0000443	hsa-miR-125a-5p	PFKM
MIMAT0002174	hsa-miR-484	ENO1
MIMAT0000443	hsa-miR-125a-5p	ENO1
MIMAT0002174	hsa-miR-484	AP2M1
MIMAT0003294	hsa-miR-625-5p	AP2M1
MIMAT0004702	hsa-miR-339-3p	ATP5B
MIMAT0002174	hsa-miR-484	NDUFV1
MIMAT0000420	hsa-miR-30b-5p	ATP5C1
MIMAT0000083	hsa-miR-26b-5p	CDK5R1

## Discussion

### EC and HIP at the early stage of AD pathology

AD is the single, most prevalent, irreversible cause of dementia, and has become an immense global societal concern. Generally, AD is divided into familial and sporadic cases, with the latter having no familial aggregation, with ~ 70% estimate of AD heritability associated factors [3–5]. The pathology progression of AD begins at EC and HIP based on Braak staging I-VI, relative to disease severity. Moreover, MRI studies in patients with AD also found the apparent volume losses in EC and HIP [35–37]. Atrophy in EC and HIP reflects the early pathological changes of AD, and the changes in EC and HIP provides potential markers in AD diagnosis [38–40]. In this study, the first purpose was to find the DEGs in EC and HIP, as shown in the schematic diagram (Fig. 1). We found 168 CDEGs in both EC and HIP, including 79 upregulated (Fig. 2C, D) and 89 downregulated (Fig. 2E, F) CDEGs. To further explore the potential functions of these CDEGs, GO enrichment and KEGG pathway analyses were performed. GO enrichment demonstrated these CDEGs were associated with various energy metabolism pathways, including canonical glycolysis, ATP hydrolysis-coupled proton transport, glycolytic process, and gluconeogenesis (Fig. 3A-C). In KEGG pathway analysis, CDEGs also could be enriched in oxidative phosphorylation (Fig. 3D). Endogenous reactive oxygen species (ROS) are the byproducts of oxidative phosphorylation that form as a result of inefficient oxidative phosphorylation. Studies have demonstrated that chronic oxidative stress contributes to the onset of AD and antioxidants can eliminate ROS and improve neuron survival to restore cognition in AD [41, 42]. Considering impaired brain energy metabolism and oxidative stress are implicated in cognitive decline in AD, our analysis results suggested that the contribution of these CDEGs in AD progression was worthy of further investigation.

### Hub genes involved in the energy metabolism

After that, we further identified 10 key hub genes (GPI, PYGB, PFKM, ATP5C1, ATP5B, ATP6V1E1, ALDOC, ATP6V0D1, ENO1, ATP6V1H; Fig. 4B and Table 1), and found these key hub genes were involved in energy metabolism and could be divided into two groups. The first group was glycolytic metabolism-related genes, including GPI, PYGB, PFKM, ALDOC, and ENO1. GPI is a member of the glucose phosphate isomerase protein family. In the cytoplasm, GPI works as a glycolytic enzyme [43]. However, outside the cell, GPI functions as a neurotrophic factor called neuroleukin, playing the role of a cytokine and neuroprotective factor. Knockdown of GPI in neuronal cells leads to caspase-dependent apoptosis [44, 45]. PYGB is a glycogen phosphorylase that is predominantly expressed in the brain but also expressed in several types of cancer [46, 47]. The brain PYGB functions as an enzyme that metabolizes glycogen to provide energy for an organism in an emergency state [48, 49]. PFKM, ALDOC, and ENO1 are also key regulatory enzymes of the glycolytic cycle. PFKM is a muscle type phosphofructokinase (PFK) involved in the conversion of fructose-6-phosphate to fructose-1,6-diphosphate [50, 51]. AD is a progressive neurodegenerative disorder characterized by misfolded Aβ, aggregated Aβ deposition causes impairments in brain regions responsible for learning and memory, and accumulation of Aβ in the brain is the primary influence driving AD pathogenesis and strongly correlated with the onset of AD [52, 53]. A study has shown that virgin olive oil upregulated the gene expression of PFKM to protect against the Aβ-induced cytotoxicity and oxidative stress by enhancing energy metabolism in vitro [54]. ALDOC catalyzes the reversible aldol cleavage of fructose-1,6,-biphosphate and fructose-1-phosphate to dihydroxyacetone phosphate and either glyceraldehyde-3-phosphate or glyceraldehyde [55, 56]. ALDOC is highly expressed in some tumor cells [57, 58], and cerebral spinal fluid (CSF) ALDOC is also expressed markedly higher after traumatic brain injury (TBI) [59]. ENO1, also known as 2-phospho-D-glycerate hydrolase, is a glycolytic enzyme that is expressed in most tissues and responsible for the conversion of 2-phosphoglyceric acid to phosphoenolpyruvic acid in the glycolytic pathway. A previous study using Redux proteomics reported dysregulation of ENO1 in cases of mild cognitive impairment (MCI) and is associated with modified hippocampus proteins and malfunction, indicating that inactivation of ENO1 leads to the development of AD from MCI [60].

The other five key hub genes were associated with ATP synthesis and cellular transport. ATP5C1 and ATP5B encode a subunit of mitochondrial ATP synthase. It has been reported that the mitochondrial ATP synthase dysfunction associates with AD progression, proven by many studies [61–63], and the mitochondrial ATP synthase could also act as a drug target for aging and dementia [64]. Moreover, ATP5C1 and ATP5B were proved as hub genes in AD progression [14, 65, 66]. ATP6V1E1, ATP6V0D1, and ATP6V1H encode a component of vacuolar ATPase (V-ATPase), which mediates acidification of eukaryotic intracellular organelles [67–69]. V-ATPase is ATP-driven proton pumps which function to acidify intracellular compartments, and V-ATPase dependent acidification is necessary for intracellular processes such as protein sorting, intracellular membrane trafficking, protein degradation, and neurotransmitter uptake [69]. It is essential to maintain a highly acidic pH in lysosomes lumen in order to perform its digestive function [70]. Lysosomal pH gradients are maintained by V-ATPase, and the lysosomal system in neurons is easily affected when lysosomal hydrolysis is impaired. Thus, the dysfunction of V-ATPase would indeed affect lysosomal acidification and disrupts its clearance of substrates, likely to lead to failure in autophagy in AD [71, 72].

It is confirmed that AD is a neurodegenerative disease that not only impairs cognitive function but also disturbs energy, glucose, lipid metabolism [22, 73]. Impaired functioning of the glycolytic pathway would indeed weaken the integrity of astrocytic-neuronal partnership, impair the brain homeostasis, and also perturbed amyloid clearance [74]. Metabolic deficits of glucose availability and mitochondrial function are well-known hallmarks in the aging brain and AD [75]. Brain metabolic dyshomeostasis plays a pivotal role in AD pathology; hence, the novel trends of AD therapy are focused on energy metabolism and regulation, including ketogenic diet [76], pharmacological, lifestyle interventions [77], and has promoted the development of some new drugs [78]. Energy and glucose metabolism alterations occur at the early stage of AD and strongly influence the progression of AD [79, 80]; our findings revealed that the functions of these 10 key hub genes are mainly to maintain the metabolic homeostasis. However, we still need more evidence to further prove that the dysregulation of these energy metabolism-related genes in EC and HIP could contribute to the early progression of AD.

### Crosstalk between miRNAs and mRNAs

miRNAs are a group of small and non-coding RNAs, each consisting of only 20–22 nucleotides [81]. miRNAs regulate more than 60% of protein expression and are associated with many neurodegenerative diseases. Accumulating evidence indicates that dysregulation of specific miRNAs involved in key regulatory genes is associated with pathogenesis and progression in AD; therefore, miRNAs-mediated regulation provides a new target of significant therapeutic prospects [16, 17]. miRNAs are stable enough in biological fluids such as the serum, plasma, and CSF; thus, their analysis in body fluids is a relatively simple, safe, and noninvasive approach [82, 83]. Analysis of miRNAs in body fluids of patients with AD seems to be useful, and circulating miRNAs is as reliable to serve as potential biomarkers in AD diagnosis [12, 84–86].

As shown in the schematic diagram of Fig. 1, to further explore the blood-related miRNAs, which could act as biomarkers in AD, the predicted DEmiRNAs from EC and HIP were intersected with the DEmiRNAs from blood samples, and a total of 55 CDEmiRNAs were identified (Fig. 4C, D). Then, 59 target genes of 55 CDEmiRNAs were further identified. Moreover, we found 10 target genes, including CALM1, AP2M1, NDUFV1, ENO1, PFKM, ATP5B, ATP5C1, GPI, ME3, CDK5R1 (Fig. 5E and Table 2), which were mainly enriched in AD, carbon metabolism, glycolysis/gluconeogenesis, and Huntington's disease, as revealed in the KEGG pathway analysis (Fig. 5D). Intriguingly, ENO1, PFKM, ATP5B, ATP5C1, and GPI also belonged to the 10 key hub genes (Fig. 4B and Table 1).

After construction of an miRNA–mRNA network, we found these 10 target genes to be regulated by hsa-miR-4659a-3p, hsa-miR-625-5p, hsa-miR-484, hsa-miR-125a-5p, hsa-miR-339-3p, hsa-miR-30b-5p, hsa-miR-378a-5p, hsa-miR-30a-5p, and hsa-miR-26b-5p (Fig. 5F and Table 3). In these 9 miRNAs, the expression level of hsa-miR-26b-5p was downregulated in the serum of patients with AD, and identified as the key miRNA associated with AD [86, 87], and a meta-analysis of gene expression data predicted the dysregulation of hsa-miR-30a-5p in the HIP of brains with AD [88]. We suspected that at the early stage of AD progression, the disease-associated miRNAs in blood or other body fluid could be transported and released into the brain tissues, such as EC and HIP. Then, these miRNAs dysregulated the target gene expression to disturb the metabolism homeostasis of brain tissues. Thus, these miRNAs, such as has-miR-26b-5p and has-miR-30a-5p, also have the potential to act as diagnostic biomarkers. However, more evidence of these miRNAs functions and regulation mechanisms needs to be explored further in future studies.

## Conclusions

Our study identified 10 key hub genes in EC and HIP of patients with AD, discovered to be involved in the glycolytic pathway or ATP metabolism, suggesting the metabolic dyshomeostasis contributed to the early AD progression. Moreover, after the construction of an miRNA–mRNA network, we identified 9 blood-related miRNAs, which regulated 10 target genes from the KEGG pathway, indicating that these miRNAs, such as has-miR-26b-5p and hsa-miR-30a-5p, had potential to act as diagnostic biomarkers. However, this research is only processed bioinformatics mining, our analysis results need to be verified by more samples especially those at the early stage of AD, and also be investigated in more basic and clinical studies in future.

## Supplementary Information

**Additional file 1: Fig. S1.** Maximal clique centrality (MCC), density of maximum neighborhood component (DMNC), and maximum neighborhood component (MNC) algorithms in the cytoHubba plugin were used to screen 15 overlapping hub genes (A). Visualization of the protein–protein interaction (PPI) network of hub genes (B). Nodes are colored according to the average |Log2FC(Fold Change)| ratio, with red representing upregulated nodes and green representing downregulated nodes.

**Additional file 2: Supplementary Data 1.** List of 2146 DEGs in the EC samples.

**Additional file 3: Supplementary Data 2.** List of 1189 DEGs in the HIP samples.

**Additional file 4: Supplementary Data 3.** List of 168 CDEGs in EC and HIP, including 79 upregulated CDEGs, and 89 downregulated CDEGs.

**Additional file 5: Supplementary Data 4.** 1047 predicted downregulated DEmiRNAs of 79 upregulated CDEGs.

**Additional file 6: Supplementary Data 5.** 832 predicted upregulated DEmiRNAs of 89 downregulated CDEGs.

**Additional file 7: Supplementary Data 6.** 140 dysregulated DEmiRNAs in the blood of patients with AD, including 58 downregulated DEmiRNAs and 82 upregulated DEmiRNAs.

**Additional file 8: Supplementary Data 7.** List of 55 CDEmiRNAs, including 30 downregulated CDEmiRNAs and 25 upregulated CDEmiRNAs.

**Additional file 9: Supplementary Data 8.** 30 upregulated target genes of 30 downregulated CDEmiRNAs.

**Additional file 10: Supplementary Data 9.** 29 downregulated target genes of 25 upregulated CDEmiRNAs.

## Data Availability

All data analyzed in this work are included in the published article and its supplementary information files.

## Abbreviations

AD: Alzheimer’s disease
EC: Entorhinal cortex
HIP: Hippocampus
miRNAs: MicroRNAs
DEGs: Differentially expressed genes
CDEGs: Common DEGs
DEmiRNAs: Differentially expressed miRNAs
CDEmiRNAs: Common DEmiRNAs
APP: Amyloid precursor protein
PS1: Presenilin-1
PS2: Presenilin-2
APOE: Apolipoprotein E
GEO: Gene Expression Omnibus
GO: Gene ontology
BP: Biological process
CC: Cellular component
MF: Molecular function
PPI: Protein–protein interactions
MCODE: Molecular complex detection
MCC: Maximal clique centrality
DMNC: Density of maximum neighborhood component
MNC: Maximum neighborhood component
ROS: Reactive oxygen species
CSF: Cerebral spinal fluid
