# Supplementary figures and images for: Bioinformatics analysis of differentially expressed genes and identification of an miRNA–mRNA network associated with entorhinal cortex and hippocampus in Alzheimer’s disease
Source: Hereditas. 2021 Jul 9;158:25. doi: 10.1186/s41065-021-00190-0 (PMC8272337; doi:10.1186/s41065-021-00190-0)

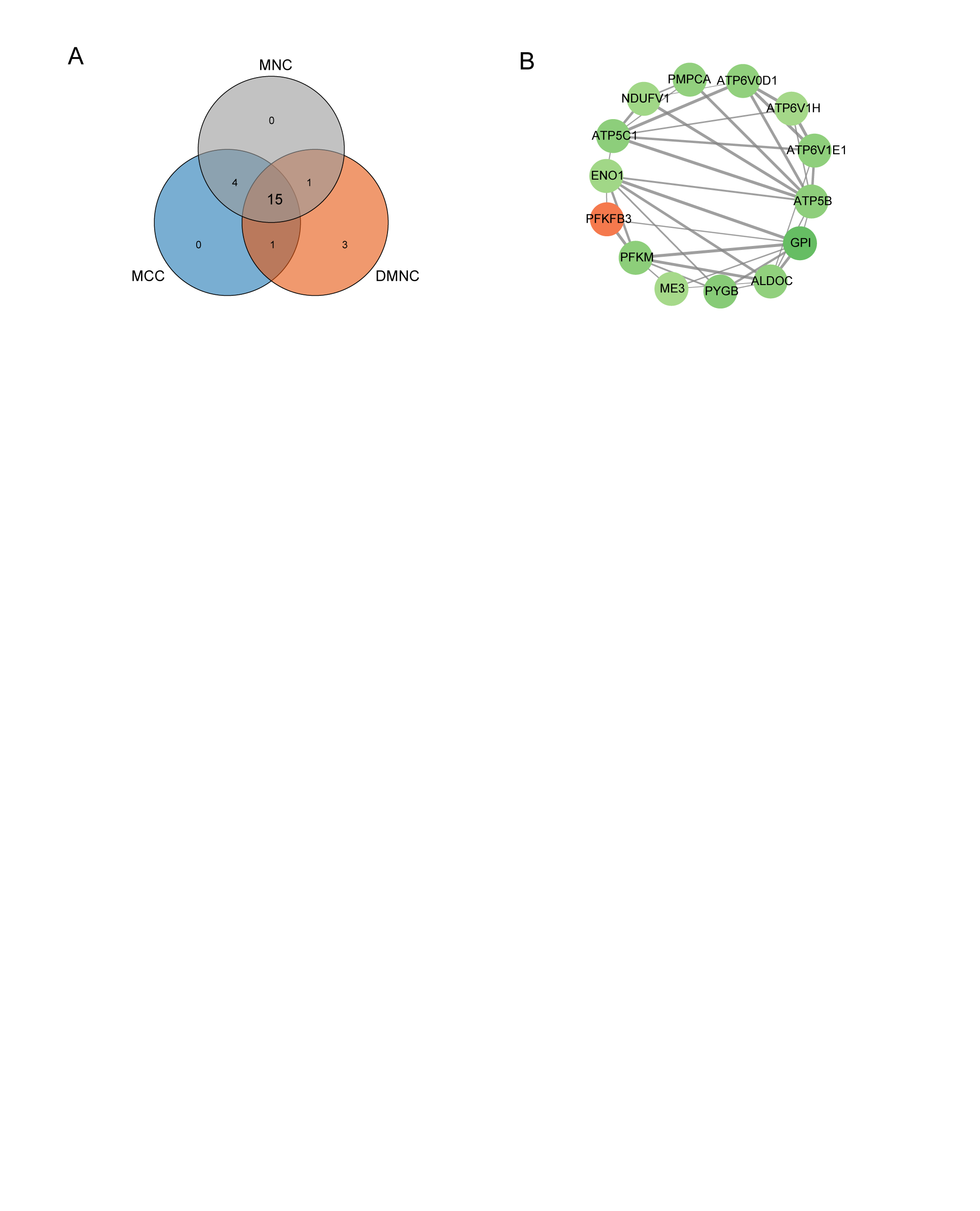

Supplement: Supplementary file 1 — Additional file 1: Fig. S1. Maximal clique centrality (MCC), density of maximum neighborhood component (DMNC), and maximum neighborhood component (MNC) algorithms in the cytoHubba plugin were used to screen 15 overlapping hub genes (A). Visualization of the protein–protein interaction (PPI) network of hub genes (B). Nodes are colored according to the average |Log2FC(Fold Change)| ratio, with red representing upregulated nodes and green representing downregulated nodes. [file 41065_2021_190_MOESM1_ESM.tif]
